# Evaluation of Some Quality Parameters of Pumpkin Seeds and Oil After Roasting with Marjoram

**DOI:** 10.3390/foods14020172

**Published:** 2025-01-08

**Authors:** Mariola Kozłowska, Małgorzata Ziarno, Katarzyna Zawada, Hanna Kowalska, Dorota Derewiaka, Małgorzata Chobot, Iwona Ścibisz

**Affiliations:** 1 Department of Chemistry, Institute of Food Sciences, Warsaw University of Life Sciences-SGGW (WULS-SGGW), 02-776 Warsaw, Poland; 2 Department of Food Technology and Assessment, Institute of Food Sciences, Warsaw University of Life Sciences-SGGW (WULS-SGGW), 02-776 Warsaw, Poland; malgorzata_ziarno@sggw.edu.pl (M.Z.); dorota_derewiaka@sggw.edu.pl (D.D.); iwona_scibisz@sggw.edu.pl (I.Ś.); 3 Department of Organic and Physical Chemistry, Faculty of Pharmacy, Medical University of Warsaw, 02-097 Warsaw, Poland; katarzyna.zawada@wum.edu.pl; 4 Department of Food Engineering and Process Management, Institute of Food Sciences, Warsaw University of Life Sciences-SGGW (WULS-SGGW), 02-776 Warsaw, Poland; hanna_kowalska@sggw.edu.pl (H.K.); malgorzata_chobot@sggw.edu.pl (M.C.)

**Keywords:** acid value, antioxidant activity, *Cucurbita pepo* L., fatty acid composition, peroxide value, specific UV extinctions

## Abstract

Consumers include pumpkin seeds in their diet as a snack in raw form or minimally processed by roasting. This process enables the seeds to develop a characteristic aroma and color. Herbs and spices are also distinguished by a pleasant and delicate aroma. Among them, marjoram is particularly suited to drying, retaining its flavor better than other dried herbs. Marjoram can be used to impart flavor and aroma to food products and extend their shelf life because it can prevent lipid autoxidation. In this study, pumpkin seeds (*Cucurbita pepo*) were roasted with and without dried marjoram at 110 and 160 °C for 10 and 30 min, after which the oils were extracted. The results showed that with increasing temperature and roasting time, the moisture content and water activity of pumpkin seeds decreased. Furthermore, roasting pumpkin seeds with marjoram, particularly at 110 °C, enriched their aroma profile with terpenes characteristic of the marjoram aroma. Whether pumpkin seeds were roasted with or without marjoram, the fatty acid composition of the oils obtained was dominated by palmitic, stearic, oleic, and linoleic acids. However, the presence of marjoram during pumpkin seeds roasting resulted in lower peroxide values and specific extinction coefficients K_232_ and K_270_ in the oils obtained compared to their counterparts roasted without this spice. In addition, all the oils showed the ability to scavenge DPPH^·^ radicals and were characterized by a higher proportion of yellow (positive value of the b* parameter) and green (negative value of the a* parameter) color. In comparison with the oil extracted from unroasted pumpkin seeds, the oil obtained after roasting exhibited a lower chlorophyll and a higher carotenoid content. Thus, roasting pumpkin seeds with spices may enrich their aroma profile with additional components, and the oils obtained may be characterized by better quality parameters.

## 1. Introduction

**Figure 1:**
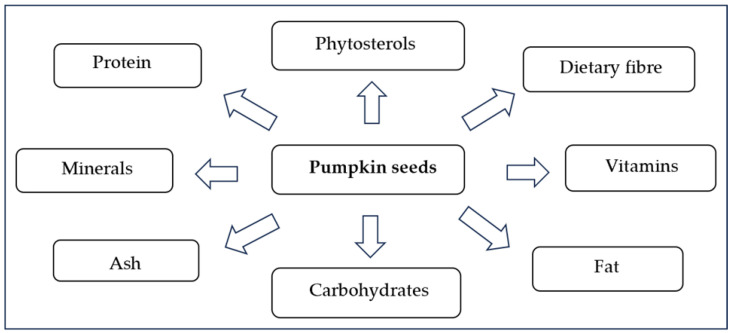
Nutritional composition of pumpkin seeds.

The pumpkin is a seasonal plant that belongs to the Cucurbitaceae family. There are three main pumpkin species used worldwide: *Cucurbita pepo* (common pumpkin), *Cucurbita moschata* (musk pumpkin), and *Cucurbita maxima* (giant pumpkin). The pumpkin’s edible parts are the fruit, seeds, leaves, and flowers. Pumpkin seeds contain many nutrients including lipids, proteins, carbohydrates, and vitamins (Figure 1) [1,2]. They also provide lecithin and bioactive molecules including tocopherols, *β*-carotene, lutein, phytosterols, and minerals [3,4]. They exhibit medicinal properties and have long been used in folk medicine for deworming children and adults, for liver and prostate diseases, and for their diuretic effect [5,6]. Pumpkin seeds also have antioxidant, anticancer, and anti-inflammatory effects [4,7]. These seeds may be eaten right after being hollowed out of the pumpkin or after they have been dried. They are perfect as an addition to salad dressings, casseroles, baked goods, and cereal mixes. They can also be ground and added to bread dough to improve its texture or used as an ingredient in various cakes instead of or in combination with nuts. Moreover, pumpkin seeds are consumers’ favorite snacks in many countries, especially when they are pre-roasted [8].

Roasting improves their sensory properties. They become more crumbly and tender, with a characteristic taste and smell [5]. After carrying out this process, it is possible to change the functional properties of the proteins contained in food products and degrade the structure of polysaccharides, thus improving the food’s digestibility, stability and shelf life [9]. The roasting process also ensures the microbiological safety of such a product, and by inactivating factors such as toxins and allergens, it improves its nutritional value [10]. Roasting seeds can modify the phenolic profile and, in some cases, improve their health properties by increasing their antioxidant capacity [11]. This process may also intensify cell damage, thus improving the extractability of the chemical components it contains and the efficiency of the extraction process [12]. Roasting is the most common thermal treatment of oilseeds before oil extraction, used to increase the yield process and modify the resulting oil’s nutritional quality and shelf life.

Pumpkin seed oil is known and appreciated in Southeastern European countries, mainly Austria, Slovenia, Croatia, and Hungary, as well as some North African and Middle-Eastern countries [13]. It is usually obtained without organic solvents during the pressing process of raw or roasted pumpkin seeds. The oil obtained from raw seeds retains its specific aroma and flavor, while that obtained from previously roasted seeds acquires a distinct nutty flavor and aroma [14]. In pumpkin seed oil, the main lipid fraction is triacylglycerols, with monoacylglycerols, diacylglycerols, and free fatty acids present in small amounts. In turn, the non-glycerol fraction includes both free and esterified sterols, phospholipids, hydrocarbons such as squalene, tocochromanols, and compounds that determine the characteristic color—chlorophylls and carotenoids [15]. The predominant fatty acids are those of an unsaturated nature beneficial to human health, mainly linoleic (C18:2) and oleic acids (C18:1), belonging to PUFA and MUFA, respectively. These fatty acids are responsible for many health-promoting properties and may play a significant role in preventing cardiovascular diseases [16,17]. However, the high content of unsaturated fatty acids in vegetable oils makes them more susceptible to oxidation processes. This can adversely affect their nutritional properties and lead to a deterioration in quality, which is indicated by sensory indicators such as taste, color, or smell, as well as physicochemical indicators such as acid value or peroxide value and fatty acid composition. The acid value determines the amount of free fatty acids in the oil. A high value indicates the processes of hydrolysis and decomposition of fats, which means a deterioration in the functional and taste properties of the oil. In turn, the peroxide value allows for measuring the amount of hydroperoxides formed in the oil during lipid oxidation. It is a key indicator of the oxidative stability of the oil. Also, K_232_ and K_270_ are parameters used to assess the degree of oxidation of oils, providing information on the content of primary and secondary oxidation products resulting from fat degradation. The presence of chlorophylls, which are strong photosensitizers in oxidation processes of unsaturated bonds of lipids, may also negatively impact the oxidative stability of oil during storage and can accelerate their oxidation. One way to improve the quality of oils and high-fat products can be using natural antioxidants, of which spices and herbs are a rich source [18,19,20,21]. Their effectiveness as antioxidants is comparable to, and often even superior to, synthetic antioxidants. Attempts are also being made to use spices and herbs in roasting seeds to increase their functional value as snacks that would be readily consumed both in daily life and while traveling. Among spices and herbs, marjoram (*Origanum majorana* L.) is particularly suitable for drying because it retains its flavor better than other dried herbs. It is a spice with a wide range of culinary and medicinal uses. It has many beneficial properties due to the presence of essential oils, flavonoids, and other bioactive substances. Marjoram has antibacterial, anti-inflammatory, and antioxidant properties, protecting the body from oxidative stress and oils from oxidation [22,23]. The incorporation of marjoram during the roasting of pumpkin seeds can give them a pleasant aroma and taste and contribute to enriching the sensory profile of the final product. Furthermore, the presence of antioxidant compounds in marjoram can counteract the formation of harmful substances, such as hydroperoxides, and thus reduce the peroxide value and limit the formation of secondary lipid oxidation products, which directly affects the quality of the oil.

The aim of this study was to determine selected physicochemical properties of roasted and unroasted pumpkin seeds (moisture content, water activity, color, and volatile compounds) and the oil obtained from them (fatty acid composition, acid and peroxide value, K_232_ and K_270_ extinction coefficients, color, and antioxidant activity) by subjecting pumpkin seeds to roasting at 110 and 160 °C for 10 and 30 min with and without the addition of dried marjoram.

## 2. Materials and Methods

### 2.1. Reagents

2,2-diphenyl-1-picrylhydrazyl (DPPH) and Trolox ((±)-6-hydroxy-2,5,7,8-tetramethylchromane-2-carboxylic acid) were supplied by Sigma-Aldrich (St. Louis, MO, USA). A certified fatty acids methyl ester (FAME) reference standard mixture (37 fatty acids from C4 to C24) was obtained from Supelco (Bellefonte, PA, USA). Other chemicals and solvents (potassium hydroxide, potassium iodide, sodium thiosulfate, anhydrous magnesium sulphate, starch soluble, phenolphthalein, ethanol, methanol, diethyl ether, chloroform, acetic acid (glacial), *n*-hexane, and isooctane) were of analytical grade. They were used as received without further purification. They were obtained from Avantor Performance Materials (Gliwice, Poland).

### 2.2. Materials

Hulled pumpkin seeds (*Cucurbita pepo* L.) and dry marjoram (*Origanum majorana* L.) were purchased from the store with organic products and healthy food in Warsaw (Poland). These raw materials were appropriately packaged and labelled with the information that these were ingredients from certified organic farming. In addition, the packaging provided product details, including recommendations for storage conditions and expiration date. Before roasting, pumpkin seeds were divided into nine portions (80 g each). One portion was kept unroasted (control), and the other eight portions without and with marjoram at a weight ratio of 10:1 (*w*/*w*) were placed in Petri dishes and roasted at 110 °C and 160 °C for 10 and 30 min using a forced-air dryer (SUP-65W, Wamed, Warsaw, Poland). After roasting, roasted dry marjoram and pumpkin seeds were separated by using a sieve. Then, they were crushed and ground in an electric coffee grinder into a fine powder, which was used to evaluate their selected physicochemical parameters and for oil extraction.

### 2.3. Moisture Content and Water Activity

Moisture content in the pumpkin seeds before and after roasting was determined by using the AOAC Standard [24]. In brief, 5 g of pumpkin seeds were placed in a dry cup in an oven regulated at 105–110 °C for 3–4 h. After heating, the cup was removed, put in a desiccator for 30 min, and weighed. This step was repeated until a constant moisture content weight was obtained. Water activity was determined in 3 repetitions using the Hygroscope DT2 (Rotronic AG, Basserdof, Switzerland) at 25 ± 1 °C.

### 2.4. Color

The color of unroasted and roasted pumpkin seeds and oils obtained from them was measured by the reflectance method using a Konica Minolta CR-A70 chromameter (Konica Minolta, Osaka, Japan) in the CIELAB model using color coordinates such as L*, a*, and b*. L* indicates the lightness of the color in the range of 0–100 (value 0 is dark and 100 is light), a* indicates red (positive value) or green (negative value), and b* indicates yellow (positive value) or blue (negative value). The final result was the arithmetic mean of 12 measurements. A standard D65 light source, a 2° observer, and a 5 mm measuring aperture were used. Before the measurement, the equipment was calibrated using white and black plates, respectively. The color parameters of the oil samples were measured by applying about 50 mL of oil to a glass so-called watch glass, and four results were recorded, each as an average of three measurements by changing the sample scanning locations using a head. The color difference between the roasted samples of pumpkin seeds (between oils obtained from roasted pumpkin seeds) and the control sample of unroasted pumpkin seeds (oil obtained from unroasted pumpkin seeds) was calculated from the equation ΔE = [(L*_c_ − L*)^2^ + (a*_ c_ − a*)^2^ + (b*_c_ − b*)^2^]^1/2^, where L*_c_, a*_c_, and b*_c_ are the color parameters of the control samples and L*, a*, and b* are the color parameters of the roasted pumpkin seed samples and oils obtained from them.

### 2.5. Volatile Compounds of Pumpkin Seeds

For the analysis of each volatile, about 4 g of ground pumpkin seeds was transferred to glass vials (20 mL) and closed with silicone septa. Sampling was performed using a DVB/CAR/PDMS fiber (1 cm long) at a 40 °C temperature, and the absorption time was 10 min by the SPME system. The fiber was inserted into the GC/MS injector, where desorption after 2 min took place (in splitless mode). The apparatus used in the experiment was GCMS-QP2010 (Shimadzu, Kyoto, Japan). The separation of aroma compounds was performed using Stabilwax (30 m × 0.25 mm × 0.25 µm, polyethylene glycol) by Restek. The carrier gas was helium, and the column flow was 0.64 mL/min. The column temperature program was as follows: initial temperature 40 °C (for 10 min) at a rate of 5 °C/min to 200 °C and 220–250 °C at a rate of 20 °C/min, holding time at 250 °C for 5 min. The total time of the analysis was 49.5 min. The interface and injector temperatures were 250 °C. The ion source temperature was 250 °C. Mass spectra were acquired in the electron impact mode (70 eV) using a full scan with a mass acquisition range of 40–450 *m*/*z*. Identification of aroma compounds was made by comparison of the mass spectrum obtained with spectra in reference libraries (NIST/SPA/NIH Mass Spectral Library, version 2.0, Faircom Corporation, Sandy, UT, USA), and from literature data. All analyses were performed in duplicate. The results are shown as the percentage of each compound in the total profile of the aroma compounds.

### 2.6. Oil Extraction

Thirty grams of grounded unroasted and roasted pumpkin seeds was extracted with *n*-hexane (250 mL) using a Soxhlet extractor for 6 h at 70 °C. After extraction, the hexane-oil mixture was passed through a layer of anhydrous magnesium sulphate placed over a Whatman filter paper in a funnel. Then, the solvent was removed by evaporation under reduced pressure using a rotary evaporator Rotavapor R-215 (BUCHI Labortechnik AG, Flawil, Switzerland) at 40 °C. The oil samples were weighed, closed under a nitrogen stream, and stored at −20 °C until further analysis. The percentage of total extractable oil was calculated.

### 2.7. Fatty Acid Composition

The fatty acid composition of the pumpkin oil samples was analyzed by gas chromatography (GC) after derivatization to fatty acid methyl esters with a 2 M methanolic potassium hydroxide solution according to the ISO 12966-2:2017 standard method [25]. A Shimadzu GC-17A (Kyoto, Japan) gas chromatograph equipped with a flame ionization detector (FID) and a BPX-70 capillary column (length 30 m, i.d. 0.22 mm, film thickness 0.25 µm; Melbourne, Australia) were used. The analysis was performed with nitrogen (1.0 mL/min) as a carrier gas at the following temperature program: 60 °C held for 1 min, after which the temperature was increased to 170 °C at a rate of 10 °C/min and from 170 to 230 °C at a rate of 3 °C/min. The temperature was kept at 230 °C for the subsequent 15 min. The injector (split ratio of 100:1) and detector temperatures were set at 225 °C and 250 °C, respectively. Individual fatty acids were identified by comparing their retention times with a certified fatty acid methyl esters (FAME) mix and quantified as a percentage of the total fatty acids.

### 2.8. Acid Value

The acid values (AV) were determined according to ISO standard methods (660:2020) [26] by titration of oil samples dissolved in a mixture of ethanol–diethyl ether (1:1; *v*/*v*) with 0.1 M ethanolic potassium hydroxide solution using phenolphthalein indicator until the pink color persisted for at least 10 s. The results of AV were expressed as mg KOH per gram of oil sample (mg KOH/g) and were calculated according to the equation: AV = (V × 5.611)/m, where V is the volume (mL) of sodium hydroxide titrant used and m is the mass of the oil sample (g).

### 2.9. Peroxide Value

The peroxide value (PV) was determined under ISO standard methods (3960:2012) [27] by titration of oil samples dissolved in a mixture of chloroform–glacial acetic acid (2:3; *v*/*v*) in the presence of saturated potassium iodide solution and starch as an indicator with 0.02 M sodium thiosulphate solution from a purple to a slightly yellow or colorless endpoint. The results of PV are shown in miliequivalent of active oxygen per kg of fat sample (meq O_2_/kg) and were calculated according to the following equation: PV = (V − V_0_) × c/m, where V and V_0_ are the volume (mL) of sodium thiosulphate exhausted by the test sample and blank, respectively, m is the mass of the oil sample (g), and c is the concentration of sodium thiosulphate (mM).

### 2.10. Specific Extinction Coefficients

The specific extinction coefficients (K_232_ and K_270_) were determined according to the ISO standard method (3656:2011) [28]. A 1% oil solution in cyclohexane was prepared (0.1 g of oil in 10 mL of cyclohexane), and absorbances were measured at 232 and 270 nm using a UV/VIS double-beam scanning spectrophotometer (Shimadzu, Kyoto, Japan). K_232_ and K_270_ were calculated using the following equation: K_λ_ = E_λ_/c × s, where K_λ_ is the specific extinction coefficient at wavelength λ, E_λ_ is measured absorbance at wavelength λ, c is the concentration of the oil solution (g/100 mL), and s is cuvette thickness (cm).

### 2.11. Antioxidant Activity

An 800 μL volume of a DPPH stock acetone solution (2.09 mmol/L) was added to 50 μL of oil samples and mixed. The mixture was kept in darkness for 20 min. Then, EPR spectra were recorded using a MiniScope MS200 spectrometer (Magnettech GmbH, Berlin, Germany) with the following set of parameters: central field 330 mT, sweep range 9.9 mT, sweep time 20 s, microwave power 6 mW, modulation amplitude 0.15 mT. The scavenging effect of oil samples on the DPPH radicals was calculated based on the decrease in the EPR signal intensities of the oil samples compared to the signal of a control sample where hexane was used instead of oil. The results were expressed as Trolox equivalents (μmol per mL of oil) using the standard calibration curve.

### 2.12. Chlorophylls and Carotenoids Content

The oil samples were dissolved in cyclohexane, and their absorbance was measured at 670 and 470 nm for the chlorophylls and carotenoids, respectively. The values obtained are expressed as mg of pheophytin a (chlorophylls) and lutein (carotenoids) per kg of oil [29] using the following equations:

[Chlorophylls] mg/kg = A_670_ × 10^6^/613 × 100 × S  [Carotenoids] mg/kg = A_470_ × 10^6^/2000 × 100 × S

where A is the absorbance and S is the thickness of the spectrophotometer cell (1 cm).

### 2.13. Statistical Analyses

All the results are reported as an arithmetic average, and the standard deviation was calculated from the replicates. Statistical analyses were performed using the Statgraphics Plus 4.0 package (Statgraphics Technologies, Inc., The Plains, VA, USA). The differences between the mean scores were determined using the analysis of variance (ANOVA). The differences between the means of multiple groups were analyzed using a two- or three-way ANOVA with Tukey’s multiple-range tests at *p* = 0.05.

## 3. Results and Discussion

### 3.1. Moisture Content and Water Activity

Food’s moisture content significantly impacts its sensory characteristics, including taste, texture, appearance, shape, and shelf life. An increased amount of water in a product also increases its susceptibility to microorganisms that can cause spoilage, making it unfit for consumption as intended. In the case of seeds, this is an important parameter determining their quality, durability, shelf life, and resistance to bacterial contamination. It is related to the maturity of the seed, the optimum time for harvesting, the method of drying, and the presence of mechanical damage. Furthermore, seeds with a higher moisture content may also yield less oil than seeds with a lower content of this parameter [30]. The moisture content of pumpkin seeds at harvest is high (30–35% wet basis), so they must be dried for storage, transport, and processing [31]. To obtain a good-quality product, they are often subjected to a roasting process. For roasted pumpkin seeds, the moisture content should not exceed 6%. Based on the data in Table 1, the moisture content in unroasted and roasted pumpkin seeds did not exceed 6%. Similar results were observed in studies conducted by Petkova and Antova [32] and Ahmed et al. [33]. An increase in the roasting temperature from 110 to 160 °C caused a decrease in the moisture content of pumpkin seeds from 4.30% to 1.11%. A decrease in the moisture content of roasted pumpkin seeds was also evident when the roasting time was increased from 10 to 30 min. On the other hand, using dried marjoram during the roasting of pumpkin seeds had no significant effect on their moisture content when the values of this parameter were compared to pumpkin seeds roasted under the same conditions but without marjoram (*p* < 0.05). However, the obtained numerical values were slightly higher when marjoram was used during roasting. The results presented in this study are comparable to those previously reported by Mohammadi-Moghaddam et al. [34], who roasted sunflower seeds with components of the sholi (coating agent) containing various spices and vegetables. The moisture content of the samples they studied ranged from 1.90% to 4.99%. The sample containing 4% paprika exhibited the lowest moisture content, while the sample containing 6% hibiscus tea demonstrated the highest moisture content.

Similar to the moisture content of seeds, water activity also affects their appearance, texture, smell, taste, and susceptibility to spoilage. Knowing this indicator makes it possible to predict the safety of the product and its stability concerning the growth of microorganisms, as well as the biochemical, physical, and enzymatic reactions that occur. Each microorganism has a specific water activity value below which it cannot grow (i.e., 0.80 minimum for bacterial growth, 0.675 minimum for yeast and molds, excluding certain xerophilic fungi) [35]. However, this does not necessarily imply their absence. The water activity in unroasted pumpkin seeds was 0.519 ± 0.07 (Table 1). The value of this parameter decreased to 0.088 ± 0.01 after roasting the pumpkin seeds at 160 °C for 30 min. Similar to the study of moisture content in pumpkin seeds, using dried marjoram during the roasting process did not have a statistically significant effect on the assessed water activity at a given temperature and roasting time. The water activity in pumpkin seeds roasted with the addition of marjoram at 160 °C for 30 min reached a value of 0.080 ± 0.01. Thus, roasting seeds can increase their sanitary quality by increasing microbiological stability and protecting them from the development of mycotoxins and the rancidity of the lipid fraction [36,37].

### 3.2. Color of Pumpkin Seeds

The roasting process affected the color of the pumpkin seeds studied (Table 1), but the change was statistically significant mainly for the a* and b* parameter values when the process was carried out at 160 °C for 30 min. Neither the increase in roasting temperature nor the presence of dried marjoram caused a significant darkening of the samples compared to the unroasted pumpkin seeds. The changes in the L* parameter values were only statistically significant when pumpkin seed samples roasted at 160 °C for 30 min were compared with samples roasted at the same time but at a lower temperature of 110 °C. At this lower temperature, pumpkin seeds were lighter than their counterparts roasted at 160 °C. The change in the values of the a* and b* parameters depended on both the applied time and roasting temperature. The parameter a*, which corresponds to red (+) and green (-) components of colors, assumed negative values for unroasted pumpkin seeds and those roasted at 110 °C. These seed samples were characterized by a more significant share of green color, regardless of the roasting time used. However, the share of red color was observed for seeds roasted at 160 °C but was more intense for samples roasted for 30 min without marjoram. The values of the parameter b*, describing yellow (+) and blue (-) components of color, assumed positive values for both roasted and unroasted seeds. Higher values of the parameter b* were characteristic of seed samples roasted at 160 °C. In particular, roasting for 30 min had a statistically significant effect on the value of this parameter compared to seeds roasted at a lower temperature. The values of the parameter b* also increased with increasing roasting time and temperature for sesame [38] and defatted roasted date seed powder [39]. The determined parameters L*, a*, and b* allowed us to calculate the absolute value of the color difference (∆E). The higher the value of this parameter, the greater the deviation from the color of the starting material. According to the studies of Francis and Clydesdale [40], only when ΔE > 3 can the color difference be perceived by the human eye. The calculated ∆E value was below 3 for the two pumpkin seed samples, indicating that the color difference was not noticeable compared to the unroasted sample. This was observed for pumpkin seeds roasted both in the presence of marjoram and its absence at 110 °C for 10 min, although in the presence of marjoram, the ΔE value was very close to 3 and amounted to 2.95 ± 0.50. The highest ΔE values were found for pumpkin seeds roasted at 160 °C for 30 min. These values were 15.96 (roasting without marjoram) and 11.58 (roasting with marjoram), respectively. Furthermore, these seeds were also characterized by the highest values for the a* and b* parameters, indicating the greatest share of red and yellow color. Extended roasting times for sunflower seed kernels using both oven and microwave also increased the total color difference (ΔE) [41]. This may be due to the formation of compounds containing unsaturated bonds in their structure, which, by absorbing light, are involved in developing a dark color in the product due to the Maillard, Strecker, or non-enzymatic browning reaction taking place.

### 3.3. Analysis of Volatiles Pumpkin Seeds

After solid-phase microextraction, the volatile components of unroasted and roasted pumpkin seeds with or without marjoram were analyzed by gas chromatography-mass spectrometry. The results obtained were summarized and are presented in Table 2. Among the volatile compounds identified in the unroasted samples, alcohols predominated, and the presence of acetic acid was confirmed. These compounds were also identified in pumpkin seed samples roasted without marjoram at 110 °C for 10 and 30 min, respectively. Among the alcohols identified were 3-methyl-1-butanol, 1-pentanol, 1-hexanol, and 2,3-butanediol. The percentage content of this class of organic compounds at 110 °C decreased with increasing roasting time of pumpkin seeds, except for the content of 1-pentanol. A similar trend was observed when pumpkin seeds were roasted at 160 °C without marjoram. However, in this case, the 1-pentanol content decreased with increasing roasting time, and the presence of 3-methyl-1-butanol was not detected. The roasting process also resulted in a reduction in acetic acid content. However, compounds derived from lipid degradation were observed. These included two identified aldehydes, hexanal and nonanal, which are oxidation products of free fatty acids such as linoleic and linolenic acids [42,43]. If the concentration of these compounds is well above certain thresholds, an undesirable rancid aroma is produced in the roasted raw material. Conversely, they impart a slightly fruity flavor note to the roasted pumpkin seeds at lower concentrations. In addition, 3-methylbutanal, a compound resulting from Strecker degradation, namely the decomposition of the relevant amino acids into aldehydes, was also present among the aldehydes [44,45]. The concentration of 3-methylbutanal was found to be significantly higher when pumpkin seeds were roasted at 160 °C for 10 min compared to a roasting time of 30 min. Generally, an increase in the temperature of the roasting process results in a decrease in the concentration of specific alcohols and aldehydes. This is primarily attributable to the enhanced volatility of these compounds at elevated temperature.

In our study, also the content of hexanal, which comes from the oxidation of linoleic acid, was higher when pumpkin seeds were roasted at 160 °C for 10 min. It is interesting to note that the content of this aldehyde is the highest at 160 °C. An increase in temperature to 200 °C results in a decrease in its content [5]. Roasting pumpkin seeds at 160 °C also resulted in the appearance of ketones and heterocyclic compounds in their volatile aromatic profile. Higher temperatures and, in our study, longer roasting times of pumpkin seeds promoted the formation of pyrazine derivatives, including 2-methylpyrazine, 2,5-dimethylpyrazine, and 2,3,5-trimethylpyrazine. Among the pyrazines identified, 2,5-dimethylpyrazine was present in the highest amount. Its content in the volatile component profile of the investigated pumpkin seeds roasted at 160 °C for 30 min was 14.97%. This was the highest content of the compound among the twelve other chemical components identified. Pyrazines are compounds formed during the thermal processing of foods, specifically during the process of non-enzymatic browning, also known as the Maillard reaction, and contribute to the nutty aroma of roasted seeds. 2,5-Dimethylpyrazine is formed as a result of heating L-threonine, while methylpyrazine is an intermediate in the heating process of L-serine as a result of the ongoing decarbonylation process, which is then followed by dehydration [46]. According to Siegmund and Murkovic [47], most pyrazines are formed when pumpkin seeds are roasted at temperatures above 120 °C but not exceeding 160 °C. Bowman and Barringer [48] found that the final levels of pyrazines and Strecker aldehydes may be higher in dehydrated roasted seeds because the Maillard reaction is less likely to occur in a higher moisture system. Furthermore, the elevated initial moisture content of the seeds may result in diminished volatile formation during the roasting process.

Roasting pumpkin seeds in the presence of dried marjoram enriched their aroma profile with components characteristic of the marjoram aroma. This was particularly noticeable when the roasting process occurred at 110 °C. The composition of the volatile compounds of pumpkin seeds included terpinen-4-ol, *γ*-terpinene, *α*-terpinene, sabinene, *β*-phellandrene, and *p*-cymene, which are considered to be the main volatile substances present in marjoram oil [49]. The presence of α-thujene and *cis*-*β*-terpineol was also noted. The concentration of these compounds was higher with increasing roasting time of pumpkin seeds, except for the presence of *α*-thujene, *α*-terpinene, *β*-phellandrene, and *p*-cymene. However, α-pinene, α-terpineol, and sabinene hydrate were not identified. All of these compounds belong to the class of terpenes, a large group of typical plant constituents composed of isoprene units with possible further modifications. Depending on the number of isoprene units present in their composition, monoterpenes, sesquiterpenes, diterpenes, triterpenes, tetraterpenes, and polyterpenes are distinguished. These compounds exhibit antibacterial, antioxidant, anti-inflammatory, and antimicrobial properties [50,51].

Terpenes were also identified in the volatile compound profile of pumpkin seeds roasted with dried marjoram at 160 °C for 30 min, but their number was reduced by three components compared to counterparts roasted at 110 °C. The roasting of pumpkin seeds with dried marjoram at 160 °C for 30 min revealed the presence of only one compound from the group of alcohols, namely 2,3-butanediol. This chemical compound has been identified as having significant potential, with a wide range of applications, including in the production of synthetic rubber, fuel additives, foodstuffs, and pharmaceuticals [52]. Two compounds from the ketone group (2-octanone, 1-phenyl-2-propnone), heptanoic acid, and esters (hexyl formate, linalyl propionate) were also identified. Hexyl formate is a compound with a fruity, raspberry-like aroma that is found in the juice of black chokeberry (*Aronia melanocarpa* Ell.), grapes (*Vitis* spp.), strawberries (*Fragaria* spp.), or peach (*Prunus persica* L.) [53].

### 3.4. Oil Extraction Yield and Fatty Acid Composition

Extraction with organic solvents and mechanical pressing are the most commonly used methods for commercially producing vegetable oils. In the present study, pumpkin seed oil extraction was carried out by the Soxhlet method using *n*-hexane. The use of *n*-hexane allows for high levels of oil extraction in a short time. This solvent has a relatively low boiling point, allowing its easy separation from the oil by distillation and its subsequent recovery and reuse. *N*-Hexane is relatively cheap and readily available, making it a cost-effective choice compared to other solvents used for fat extraction. It enables the selective extraction of fat without interfering with other nutrients such as fiber or protein. However, it is necessary to thoroughly remove its traces from the oil before consumption to meet food safety standards. Compared to cold pressing methods, Soxhlet extraction may result in slightly lower contents of some bioactive compounds such as tocopherols in the obtained oil. The resulting mean oil extraction yields with standard deviations are shown in Table 3. They ranged from 26.32 to 34.24%. Oil from unroasted pumpkin seeds was obtained with a yield of 32.27% ± 0.48. There were no statistically significant differences (*p* > 0.05) between the yield values obtained for oils from the unroasted plant material and those roasted at a specific temperature and for an appropriate time, except for the yield value obtained for oil samples extracted from seeds roasted for 30 min in the presence of dried marjoram. These were characterized by the lowest yield value of 26.32%. The extraction yield results obtained for pumpkin seed oils are comparable to those reported by other researchers who have extracted pumpkin seed oil using alternative methods [54,55]. Singh and Kumar et al. [54] showed that the Soxhlet extraction method allowed for obtaining pumpkin seed oil with a higher yield (38.03%) than when cold press extraction (33.25%) and mechanical shaking extraction (26.36%) were used. Irnawati et al. [55] also confirmed the higher extraction yield of pumpkin seed oil using the Soxhlet method compared to hot pressing and ultrasound-assisted methods. This may be due to the longer contact of the solvent used with the starting material, its cyclic washing, and the use of higher temperatures. In turn, Hu et al. [56] showed that microwave pre-treatment can increase the yield of oil extraction from pumpkin seeds compared to their direct cold pressing method. Differences in the yield of oils obtained from seeds often depend not only on the extraction method used but also on the climate in which the raw material was cultivated, the ripening stage and the harvesting time of the seeds [57].

The pumpkin seed oils obtained consisted mainly of unsaturated fatty acids (UFA), which benefit human health (Table 3). They accounted for about 80% of the relative fatty acid content. The roasting temperature of pumpkin seeds did not result in significant differences in the UFA content of the characterized oils [13]. However, their content was slightly higher in samples roasted at 160 °C, especially when the roasting was carried out in the presence of dried marjoram. More significant differences were observed when comparing the content of monounsaturated fatty acids (MUFA) and polyunsaturated fatty acids (PUFA) in samples of oils obtained from unroasted and roasted pumpkin seeds. The analysis of the relative fatty acid content of pumpkin seeds conducted by Peng et al. [5] also showed that unsaturated fatty acids accounted for about 83% of the total fatty acids and that roasting temperature had no apparent effect on their composition. Pumpkin seed oil is a typical highly unsaturated oil with a predominance of oleic and linoleic fatty acids, although the ratio of these fatty acids depends on the variety, climate, cultivation conditions, and degree of maturity of the oilseed. Linoleic acid, which belongs to the n-6 and PUFA family, was the main fatty acid in the composition of the oil samples analyzed. This precursor of arachidonic acid represented more than 50% of the total fatty acids. Its content was higher in samples roasted at a higher temperature and with the addition of dried marjoram. The presence of this acid is important for the correct growth and development of the young organism. It is essential for proper functioning of the liver and kidneys and reduces the concentration of triacylglycerols and cholesterol in the blood serum [17]. It inhibits platelet aggregation and lowers blood pressure. The second most abundant unsaturated fatty acid in the oil samples analyzed was oleic acid, which belongs to the monounsaturated fatty acid (MUFA) class. It is very effective in reducing the risk of cardiovascular diseases and infections [58]. Its content in the tested oil samples ranged from 26.66 to 27.54% and was only slightly lower in three samples than in the sample obtained from unroasted raw material. The high level of oleic acid is nutritionally essential and gives the products high resistance during cooking and frying. Pumpkin seed oil is a rich source of both oleic and linoleic acid and can be used as a cooking and salad oil and in the production of margarine.

Saturated fatty acids, such as palmitic acid and stearic acid, were found to be prevalent in the fatty acid composition of the pumpkin seed oils examined [59,60]. The palmitic acid content ranged from 11.87 to 12.66% and the stearic acid content from 6.75 to 7.16%. These results are similar to those reported by Boujemaa et al. [61] and Siano et al. [62] for the stearic acid content of oil derived from unroasted *Cucurbita pepo* seeds but are lower in the case of palmitic acid. However, compared to the results presented by Sinkovič and Kolmanič [63], the palmitic acid content in oils from traditional cultivars was similar to the studied oil obtained from unroasted pumpkin seeds. In turn, the stearic acid content was significantly higher in oils obtained from both traditional and hybrid varieties. Compared to the oil obtained from unroasted pumpkin seeds, the oils obtained from pumpkin seeds roasted at 110 °C for 10 min with marjoram and those roasted for 30 min without the spice were characterized by a higher palmitic acid content. For stearic acid content, statistically significant differences were observed between pumpkin seed oil samples roasted at 160 °C for 10 min and those roasted for 30 min in the presence of marjoram. Regardless of whether the pumpkin seeds were roasted with or without marjoram, the predominant fatty acids in the oil samples were four acids, namely linoleic, oleic, palmitic, and stearic acid. The other acids were found in amounts not exceeding 2% [12].

### 3.5. Physicochemical Properties of Pumpkin Seed Oils

#### 3.5.1. Acid and Peroxide Values or Specific Extinction Coefficients

The chemical properties of the pumpkin seed oils analyzed are shown in Table 4. The acid and peroxide values, specific extinction coefficients (K_232_, K_270_), and antioxidant activity were determined. They are used to determine the unfavorable changes that occur in fats, especially during storage, and are treated as parameters of their quality. In general, the higher the acid and peroxide values, the lower the quality of the oil tested [64]. The acid value (AV), which defines the amount of free fatty acids in the oils studied, ranged from 2.59 to 3.62 mg KOH/g oil. These values indicate the good quality of the oils studied, as they did not exceed the maximum limit for unrefined oils of 4.0 mg KOH/g oil (2% oleic acid) according to the Codex Alimentarius [65]. In the studies by Nederal et al. [12], the free fatty acid content expressed as % of oleic acid in samples of oils extracted from roasted seeds also did not exceed the values given in the Codex Alimentarius [65]. They did not differ significantly between the pumpkin seed oils analyzed and were less than 1%. The highest acid value was found in the oil obtained from unroasted raw material (3.62 mg KOH/g oil). However, statistically significant lower values of this parameter were determined for pumpkin seed oil samples subjected to roasting at 160 °C for both 10 and 30 min. They were also characterized by lower moisture and water activity values. Roasting seeds at higher temperatures may cause a decrease in the acid value due to faster inactivation of oxidizing enzymes [66]. Lower free fatty acid values were also observed in cold-pressed pumpkin seed oil samples after roasting at 170 °C for 15 min, irrespective of the type of *Cucurbita pepo* cultivar used and one of the three pretreatments carried out on the raw material [8]. In contrast, cold-pressed oils from *Cucurbita pepo* cultivated in Morocco showed a slight increase in acid value with increasing roasting temperature from 90 to 150 °C. There was no deterioration in oil quality, as the AV values obtained did not exceed 4 mg KOH/g of oil [61]. In turn, oils pressed from *Cucurbita maxima* showed no significant difference in the acid values with increasing roasting temperature of the seeds. The presence of marjoram during the roasting of pumpkin seeds did not cause an increase in the acid value of the oil samples studied above the value obtained for unroasted raw material. The AV values did not differ statistically significantly (*p* > 0.5) between the pumpkin seed oil samples roasted at 110 °C for 10 and 30 min, without and with the addition of marjoram. However, differences were observed when roasting was carried out at 160 °C for 10 and 30 min. Regardless of time roasting, the AV values were higher for oils obtained from pumpkin seeds roasted with marjoram.

High temperature promotes the formation of free radicals, which interact with atmospheric oxygen to form hydroperoxides. These are the main products of the initial phase of lipid autoxidation. Hydroperoxides are non-volatile, odorless and relatively unstable compounds, the decomposition of which can lead to the formation of volatile aromatic compounds, which are perceived as unpleasant tastes and indicate that the food is no longer suitable for consumption [67]. Their content in oils is determined by measuring the peroxide value (PV). The PV, which indicates the presence of primary fat oxidation products, was in the range of 3.82–9.89 meq O_2_/kg oil. The value of this parameter increased with the increasing temperature and roasting time of pumpkin seeds. The lowest value was observed for oil extracted from pumpkin seeds before the roasting process, while the highest PV value was characteristic of oil whose raw material was roasted at 160 °C for 30 min. At this time, the peroxide value increased almost threefold. Lower values of this parameter were also observed in cold-pressed pumpkin seed oil samples [68]. Similarly to our study, they increased after roasting the seeds at 130 °C for 30 min, but the peroxide value was lower. In turn, the values of this parameter determined for oils extracted from pumpkin seeds by three methods, namely cold press extraction, Soxhlet extraction, and mechanical shaking extraction, ranged from 2.02 to 3.88 meq O_2_/kg of oil [54]. The highest PV value was found for oil extracted from pumpkin seeds using the Soxhlet apparatus and the lowest for cold-pressed oil. The differences in PV were influenced by the type of extraction method chosen, including the use of solvent and higher temperature. It should be emphasized that the PV values reported by Singh and Kumar [54] for pumpkin seed oil obtained using the Soxhlet apparatus were similar to those presented in our study. In addition, the presence of marjoram during the roasting of pumpkin seeds resulted in lower PV values of the analyzed oils than their counterparts obtained during the roasting process without the presence of this spice, especially when it was carried out at 110 °C. Dried marjoram leaves are often added at the end of recipes because of their specific flavor and aroma. The composition of their aroma contains organic components belonging to the terpene class (Table 2), which exhibit antioxidant properties and protect against oxidative stress in various diseases [69]. This class of compounds was also identified in the volatile composition of pumpkin seeds roasted with marjoram but was not present in their counterparts roasted without the addition of dried marjoram.

The absorption values at specific wavelengths, 232 and 270 nm, indicate the presence of conjugated dienes and trienes in the oil samples studied. The formation of these compounds is related to the oxidation of the oils and is expressed by specific extinction coefficients denoted as K_232_ and K_270_. The specific extinction coefficient K_232_ is associated with the formation of primary oil oxidation products, while K_270_ is associated with secondary oil oxidation products. According to Boujemaa et al. [61], K_232_ is used to measure the autoxidation index of the oil, while K_270_ measures the presence of conjugated dienes and trienes. In our study, the lowest values of these two coefficients were observed for oil extracted from unroasted pumpkin seeds. They were 1.82 ± 0.03 for K_232_ and 0.73 ± 0.02 for K_270_. These results are lower than those reported by Can-Cauich et al. [70] and Rezig et al. [71]. In turn, higher values of the extinction coefficients K_232_ and K_270_ were observed for oil samples obtained from pumpkin seeds roasted without marjoram. For these oils obtained from pumpkin seeds roasted at 110 °C for 10 min, the K_232_ parameter values were at the level of 2.13 ± 0.05, and values for the K_270_ parameter were at the level of 0.87 ± 0.05. For comparison, their counterparts extracted from pumpkin seeds roasted under the same conditions but in the presence of dried marjoram were characterized by lower values of both parameters. Thus, the values of the parameter K_232_ varied at 1.93 ± 0.04 and those for the parameter K_270_ at 0.81 ± 0.03. In general, the values of these two coefficients were lower when pumpkin seeds were roasted with marjoram, regardless of the roasting time and temperature used.

#### 3.5.2. Antioxidant Activity

The antioxidant activity of oils extracted from unroasted and roasted pumpkin seeds was evaluated using EPR spectroscopy with the DPPH^·^ radical assay (Table 4). All the oil samples studied showed the ability to scavenge DPPH^·^ radicals. It was highest for the oil obtained from pumpkin seeds not subjected to the roasting process and amounted to 5.44 µmol TE/g oil. The DPPH^·^ radical scavenging ability of oils obtained from pumpkin seeds roasted at 110 °C for 30 min, both without and with dried marjoram, was also at a similar level. In turn, oils extracted from pumpkin seeds roasted at 160 °C for 10 and 30 min showed lower antioxidant activity (4.40 ± 0.11 and 4.41 ± 0.27 µmol TE/g oil, respectively). Although the presence of dried marjoram during the roasting of pumpkin seeds did not cause statistically significant changes in the antioxidant activity of the oil samples studied, the values obtained were slightly higher than those attributed to their counterparts obtained from pumpkin seeds roasted without the presence of dried marjoram. This could be influenced by the presence of the same volatile compounds in the aroma profile of both dried marjoram and pumpkin seeds roasted in the presence of marjoram, especially at 110 °C. It is worth noting that extracts and etheric oils obtained from marjoram, as well as other spices and herbs of the Lamiacea family, are a source of phenolic compounds that correlate positively with their antioxidant activity. They can be successfully used as potential replacements for synthetic antioxidants such as BHA and improve the oxidative stability of studied oils [72,73]. In the studies carried out by Aktas et al. [8], it is also possible to observe slightly higher values of radical scavenging activity in oils obtained from sun-dried unroasted pumpkin seeds of one of the varieties belonging to *Curcubita pepo* compared to their roasted counterparts obtained from seeds of both unsalted and dry-salted varieties. At the same time, they showed that the oil obtained from seeds roasted after the process of wet salting was characterized by an antioxidant activity similar to that of oils obtained from sun-dried unroasted pumpkin seeds. On the other hand, Ahmed et al. [33] observed that roasting pumpkin seeds at 170 °C for 60 min contributed to an increase in total phenolics, flavonoids, and DPPH antioxidant capacity compared to raw seeds as a result of the Maillard reaction products that occur. This may also be reflected in the antioxidant activity of oils obtained from this raw material [4].

#### 3.5.3. Oil Color, Chlorophylls, and Carotenoids Content

Color is an essential feature for the consumer that determines the visual acceptability of the oil. It can be attributed to the pigments present in the oil as well as to morphological factors. With regard to the L* parameter, the extracted oils can be divided into two groups. The first group consists of oils obtained from pumpkin seeds roasted at 110 °C and not subjected to roasting, while the second group consists of oils extracted from pumpkin seeds roasted at 160 °C (Table 5).

The oils roasted at the lower temperature were darker than those roasted at the higher temperature but did not differ statistically significantly in the L* parameter from the oils extracted from unroasted pumpkin seeds and those roasted with marjoram. In turn, the oils obtained from pumpkin seeds roasted at a higher temperature in the presence of marjoram exhibited lower L* parameter values than those obtained from pumpkin seeds roasted without dried marjoram. Negative values of the a* parameter were observed for all the oil samples, indicating a more significant share of green color, with the saturation of this color being more pronounced for the pumpkin seed oils roasted without marjoram. However, this did not correspond to the chlorophyll content in these oils. The oil samples obtained from unroasted pumpkin seeds and seeds roasted with marjoram were characterized by a higher chlorophyll content than their counterparts roasted without marjoram, except for the oil obtained from pumpkin seeds roasted at 160 °C for 30 min. All the oils had positive values for the parameter b*, which indicates their saturation with yellow color, often corresponding to the carotenoid content of these oils. The carotenoid content in the oil obtained from unroasted pumpkin seeds was the lowest, but slightly higher than in the oils obtained from unroasted seeds of the three pumpkin varieties, in the range of 0.25–0.66 mg/kg [61]. When the roasting process was carried out at 110 °C, the oils obtained from pumpkin seeds roasted without marjoram were characterized by a higher content of carotenoids. In turn, roasting the seeds at 160 °C resulted in a higher content of these pigments in oils obtained from pumpkin seeds roasted with marjoram. The content of total carotenoids in oils pressed from roasted pumpkin seeds in the study by Vujasinovic et al. [68] was higher than in our study, but it was observed that extending the roasting time of the seeds to 60 min and increasing the temperature resulted in a decrease in the carotenoid content due to possible degradation of β-carotene or its polymerization.

The most significant color differences were observed in oils obtained from pumpkin seeds roasted at 160 °C, for which the ΔE was 21.92 ± 0.77 (10 min roasting) and 17.97 ± 1.87 (30 min roasting), respectively. The ΔE values of oils extracted from pumpkin seeds roasted under the same conditions but in the presence of marjoram were lower but twice as high when roasting was carried out in the presence of this spice at 110 °C.

## 4. Conclusions

The main objective of this study was to investigate the effect of roasting in the presence of dried marjoram on some quality parameters of pumpkin seeds and oils. The results showed that the roasting process decreased the moisture content and water activity of pumpkin seeds, and the presence of marjoram enriched their aroma profile with components characteristic of this spice. The oils obtained were characterized by lower values of acidity and chlorophyll content. Although the values of peroxide value and specific extinction coefficients were higher for oils obtained from pumpkin seeds after roasting than before roasting, they were lower when roasting took place in the presence of marjoram. The compounds present in marjoram and those formed during the roasting process may also have influenced the results of the analysis of the antioxidant activity of the oils obtained. Therefore, roasting in the presence of spices can improve the flavor of the seeds and the quality of the oils obtained. Nevertheless, this process is not without limitations and potential difficulties. High temperatures can lead to the degradation of the bioactive compounds present in marjoram, thereby reducing its antioxidant efficacy. Furthermore, over-roasting can result in the development of a bitter taste in the seeds and a loss of the marjoram’s sensory properties, rendering it brittle. To reduce these problems, the roasting process should be carefully planned and controlled.

## Figures and Tables

**Table 1:** Moisture content (%), water activity and color profile of unroasted and roasted pumpkin seeds.

Pumpkin Seeds		Moisture Content (%)	Water Activity	L*	a*	b*	∆E
Unroasted		5.70 ± 0.10 ^e^	0.519 ± 0.07 ^d^	68.53 ± 0.02 ^a,b^	−2.48 ± 0.04 ^a^	14.41 ± 0.24 ^a^	-
Roasted							
110 °C	10 min	4.30 ± 0.65 ^d,e^	0.396 ± 0.06 ^c,d^	69.64 ± 1.36 ^a,b^	−2.19 ± 0.04 ^a^	14.68 ± 0.77 ^a^	1.44 ± 1.25 ^a^
	10 min with marjoram	4.58 ± 0.46 ^d,e^	0.409 ± 0.00 ^c,d^	70.88 ± 1.08 ^a,b^	−2.48 ± 0.05 ^a^	15.99 ± 0.94 ^a^	2.95 ± 0.50 ^a,b^
	30 min	3.44 ± 0.44 ^b,c,d^	0.309 ± 0.00 ^b,c^	72.44 ± 1.63 ^b^	−2.35 ± 0.07 ^a^	16.61 ± 0.34 ^a^	4.53 ± 1.37 ^a,b^
	30 min with marjoram	3.83 ± 0.23 ^c,d^	0.325 ± 0.05 ^b,c^	70.88 ± 1.96 ^a,b^	−2.55 ± 0.21 ^a^	16.51 ± 0.35 ^a^	3.23 ± 1.83 ^a,b^
160 °C	10 min	1.85 ± 0.77 ^a,b^	0.145 ± 0.04 ^a^	68.93 ± 4.06 ^a,b^	0.93 ± 1.03 ^b^	20.56 ± 2.05 ^b,c^	7.66 ± 2.07 ^b,c^
	10 min with marjoram	2.59 ± 0.22 ^a,b,c^	0.214 ± 0.04 ^a,b^	71.05 ± 3.85 ^a,b^	−0.76 ± 0.50 ^a,b^	17.84 ± 1.23 ^a,b^	5.36 ± 1.01 ^a,b^
	30 min	1.11 ± 0.06 ^a^	0.088 ± 0.01 ^a^	62.04 ± 1.99 ^a^	6.20 ± 0.66 ^d^	26.08 ± 0.43 ^d^	15.96 ± 1.34 ^d^
	30 min with marjoram	1.26 ± 0.08 ^a^	0.080 ± 0.01 ^a^	62.81 ± 1.48 ^a^	3.17 ± 0.50 ^c^	22.70 ± 0.15 ^c,d^	11.58 ± 0.93 ^c,d^

Data are the means of three independent experiments ± standard deviations (n = 3). ^a–e^ Values in the same column with different lowercase letters indicate a significant difference at the 0.05 significance level (Tukey HSD).

**Table 2:** Volatile compounds (%) of unroasted and roasted pumpkin seeds.

Name	Peak Area Ratio (%)									
	Unroasted Material		Roasted Pumpkin Seeds							
			110 °C				160 °C			
	Marjoram	Pumpkin Seeds	10 min	10 min with Marjoram	30 min	30 min with Marjoram	10 min	10 min with Marjoram	30 min	30 min with Marjoram
Terpenes										
*α*-Thujene	1.08 ± 0.08 ^a^	- ^1^	-	8.94 ± 0.21 ^c^	-	5.28 ± 0.46 ^b^	-	-	-	-
Sabinene	5.88 ± 0.98 ^a^	-	-	6.17 ± 0.77 ^a^	-	8.26 ± 0.30 ^b^	-	-	-	9.11 ± 0.01 ^b^
*α*-Terpinene	11.31 ± 1.01 ^b^	-	-	7.14 ± 0.02 ^a^	-	7.18 ± 0.69 ^a^	-	-	-	-
*β*-Phellandrene	8.69 ± 0.87 ^c^	-	-	4.35 ± 0.26 ^a^	-	4.38 ± 0.66 ^a^	-	6.98 ± 0.22 ^b^	-	
*p*-Cymene	15.79 ± 1.67 ^a^	-	-	19.35 ± 0.34 ^b^	-	13.84 ± 0.54 ^a^	-	-	-	-
*α*-Pinene	0.89 ± 0.14 ^a^	-	-	-	-	-	-	-	-	7.39 ± 0.64 ^b^
*γ*-Terpinene	4.89 ± 0.93 ^b^	-	-	5.19 ± 0.06 ^b^	-	7.21 ± 0.09 ^a^	-	-	-	5.42 ± 0.80 ^b^
*cis*-*β*-Terpineol	10.75 ± 1.32 ^b^	-	-	8.99 ± 0.56 ^a,b^	-	12.91 ± 0.55 ^c^	-	-	-	8.16 ± 0.01 ^a^
*α*-Terpineol	8.12 ± 0.52	-	-	-	-	-	-	-	-	-
Terpinen-4-ol	11.75 ± 1.32 ^c^	-	-	9.13 ± 0.19 ^b^	-	10.89 ± 0.61 ^b,c^	-	3.65 ± 0.06 ^a^	-	5.10 ± 0.36 ^a^
Sabinene hydrate	1.05 ± 0.02 ^b^	-	-	-	-	-	-	5.56 ± 0.16 ^b^	-	-
**Alcohols**										
3-Methyl-1-butanol	-	6.31 ± 0.44 ^b^	11.64 ± 0.97 ^c^	-	10.62 ± 0.11 ^c^	-	-	3.76 ± 0.63 ^a^	-	-
1-Pentanol	-	11.44 ± 0.01 ^d^	9.16 ± 0.65 ^c^	2.69 ± 0.03 ^a^	13.02 ± 0.21 ^e^	2.96 ± 0.42 ^a^	9.22 ± 0.92 ^c^	-	5.10 ± 0.85 ^b^	-
1-Hexanol	-	32.16 ± 0.50 ^f^	28.12 ± 0.45 ^e^	13.96 ± 0.26 ^c^	26.94 ± 1.06 ^e^	11.42 ± 0.23 ^b^	16.69 ± 1.26 ^d^	18.65 ± 1.27 ^d^	9.13 ± 0.16 ^a^	-
2,3-Butanediol	-	16.38 ± 0.04 ^d^	20.90 ± 0.36 ^e^	6.54 ± 0.09 ^a^	16.66 ± 1.16 ^d^	5.69 ± 0.09 ^a^	15.72 ± 1.22 ^d^	11.60 ± 0.16 ^b,c^	12.67 ± 0.81 ^c^	10.26 ± 1.06 ^b^
5-Methyl-5-hexen-2-ol	-	-	-	-	-	-	2.84 ± 0.13	-	-	-
**Aldehydes**										
3-Methylbutanal	-	-	-	-	-	-	6.15 ± 0.19 ^b^	-	5.21 ± 0.09 ^a^	-
Hexanal	-	-	-	-	-	-	16.69 ± 0.91 ^c^	13.08 ± 0.81 ^b^	11.01 ± 0.72 ^a^	-
Nonanal	-	-	-	-	-	-	-	-	4.87 ± 0.11	-
**Ketones**										
2-Octanone	-	-	-	-	-	-	-	-	-	4.86 ± 0.42
1-Phenyl-2-propanone	-	-	-	-	-	-	-	1.26 ± 0.81 ^a^	1.32 ± 0.14 ^a^	0.69 ± 0.07 ^a^
2-Heptanone	-	-	-	-	-	-	1.44 ± 0.51 ^a^	-	3.61 ± 0.70 ^b^	-
**Acids**										
Acetic acid	3.35 ± 0.54 ^b^	4.05 ± 0.37 ^b^	-	-	-	0.96 ± 0.27 ^a^	-	-	-	-
2-Methylbutyric acid	-	-	-	-	7.26 ± 0.81 ^a^		7.39 ± 0.56 ^a^	-	6.58 ± 0.41 ^a^	-
4-Methylpentanoic acid	-	-	3.15 ± 0.44	-	-	-	-	-	-	-
Heptanoic acid	-	-	-	-	-	-	-	3.75 ± 0.65 ^a^	-	5.28 ± 0.40 ^b^
**Ester**										
Hexyl formate	–	-	-	-	-	-	-	-	-	22.61 ± 0.67
Ocimenyl acetate	2.16 ± 0.32	-	-	-	-	-	-	-	-	-
Linalyl propionate	-	-	-	2.39 ± 0.58 ^b,c^	-	3.01 ± 0.23 ^c^	-	1.00 ± 0.23 ^a^	-	1.85 ± 0.19 ^a,b^
**Alkanes**										
Octane	-	-	-	-	-	-	-	3.11 ± 0.71 ^b^	-	0.90 ± 0.01 ^a^
**Heterocycles**										
2-Methylpyrazine	-	-	-	-	-	-	-	-	3.84 ±0.86	-
2,5-Dimethylpyrazine	-	-	-	-	-	-	-	-	14.97 ± 1.74 ^b^	8.49 ± 0.12 ^a^
2,3,5-Trimethylpyrazine	-	-	-	-	-	-	-	-	3.48 ± 0.23 ^b^	1.52 ± 0.25 ^a^

Data are the means of three independent experiments ± standard deviations (n = 3). ^a–f^ Values within the same row with different lowercase letters indicate a significant difference at the 0.05 significance level (Tukey HSD); ^1^ not detected.

**Table 3:** Fatty acid composition (%) of oil extracted from unroasted and roasted pumpkin seeds.

Fatty Acids	Unroasted Pumpkin Seeds	Roasted Pumpkin Seeds							
		110 °C				160 °C			
		10 min	10 min with Marjoram	30 min	30 min with Marjoram	10 min	10 min with Marjoram	30 min	30 min with Marjoram
C14:0 (Myristic)	0.13 ± 0.01 ^a^	0.13 ± 0.01 ^a^	0.15 ± 0.01 ^a^	0.14 ± 0.00 ^a^	0.13 ± 0.01 ^a^	0.12 ± 0.00 ^a^	0.14 ± 0.01 ^a^	0.14 ± 0.00 ^a^	0.12 ± 0.00 ^a^
C16:0 (Palmitic)	12.09 ± 0.01 ^d^	11.87 ± 0.01 ^a^	12.66 ± 0.00 ^f^	12.25 ± 0.02 ^e^	11.95 ± 0.01 ^a,b^	11.98 ± 0.01 ^b,c^	11.96 ± 0.01 ^a,b^	11.96 ± 0.01 ^a,b^	12.08 ± 0.02 ^c,d^
C16:1 (Palmitoleic)	0.10 ± 0.00 ^a^	0.11 ± 0.00 ^a,b^	0.11 ± 0.00 ^a,b^	0.12 ± 0.01 ^b^	0.12 ± 0.00 ^b^	0.10 ± 0.00 ^a^	0.10 ± 0.00 ^a^	0.11 ± 0.00 ^a,b^	0.10 ± 0.00 ^a^
C18:0 (Stearic)	7.06 ± 0.01 ^c,d^	7.16 ± 0.01 ^d^	6.98 ± 0.01 ^b,c^	7.12 ± 0.01 ^d^	7.08 ± 0.02 ^c,d^	6.75 ± 0.01 ^a^	6.89 ± 0.01 ^b^	7.07 ± 0.02 ^c,d^	6.75 ± 0.07 ^a^
C18:1 9c (Oleic)	27.07 ± 0.03 ^c^	27.37 ± 0.01 ^e^	27.46 ± 0.01 ^f^	27.54 ± 0.02 ^g^	26.66 ± 0.01 ^a^	27.52 ± 0.01 ^f,g^	26.99 ± 0.01 ^b^	26.94 ± 0.02 ^b^	27.18 ± 0.01 ^d^
C18:2 9c 12c (Linoleic)	52.08 ± 0.07 ^c^	51.87 ± 0.01 ^b^	51.24 ± 0.01 ^a^	51.35 ± 0.01 ^a^	52.38 ± 0.02 ^d,e,f^	52.33 ± 0.01 ^d,e^	52.46 ± 0.01 ^f^	52.27 ± 0.02 ^d^	52.42 ± 0.00 ^e,f^
C18:3 9c 12c 15c (α–Linolenic)	0.21 ± 0.00 ^a^	0.26 ± 0.00 ^e^	0.24 ± 0.00 ^c^	0.25 ± 0.00 ^d^	0.28 ± 0.00 ^f^	0.21 ± 0.00 ^a^	0.25 ± 0.00 ^d^	0.26 ± 0.00 ^e^	0.22 ± 0.00 ^b^
C20:0 (Arachidic)	0.49 ± 0.01 ^b^	0.51 ± 0.01 ^b,c^	0.48 ± 0.00 ^b^	0.52 ± 0.01 ^c^	0.52 ± 0.00 ^c^	0.43 ± 0.00 ^a^	0.50 ± 0.01 ^b,c^	0.52 ± 0.00 ^c^	0.45 ± 0.00 ^a^
C20:1 9c (Eicosenic)	0.11 ± 0.01 ^c,d^	0.10 ± 0.00 ^c^	0.10 ± 0.00 ^c^	0.11 ± 0.00 ^d^	0.11 ± 0.00 ^d^	0.08 ± 0.00 ^a^	0.10 ± 0.00 ^c^	0.10 ± 0.00 ^c^	0.09 ± 0.00 ^b^
C22:0 (Behenic)	0.09 ± 0.00 ^c^	0.15 ± 0.01 ^g,h^	0.10 ± 0.00 ^d^	0.13 ± 0.00 ^f^	0.15 ± 0.00 ^h^	0.06 ± 0.00 ^a^	0.12 ± 0.01 ^e^	0.14 ± 0.00 ^g^	0.07 ± 0.00 ^b^
C24:0 (Lignoceric)	0.58 ± 0.01 ^c,d^	0.53 ± 0.01 ^a,b^	0.49 ± 0.01 ^a,b^	0.53 ± 0.00 ^b,c^	0.60 ± 0.01 ^d^	0.48 ± 0.02 ^a^	0.51 ± 0.02 ^a,b^	0.54 ± 0.00 ^b,c^	0.51 ± 0.00 ^a,b^
SFA	20.43 ± 0.11 ^c^	20.33 ± 0.05 ^c^	20.86 ± 0.04 ^d^	20.69 ± 0.05 ^d^	20.43 ± 0.06 ^c^	19.82 ± 0.05 ^a^	20.11 ± 0.07 ^b^	20.37 ± 0.04 ^c^	19.98 ± 0.09 ^a,b^
MUFA	27.28 ± 0.04 ^c^	27.58 ± 0.01 ^e^	27.67 ± 0.01 ^f^	27.77 ± 0.04 ^g^	26.89 ± 0.01 ^a^	27.70 ± 0.01 ^f,g^	27.19 ± 0.01 ^b^	27.15 ± 0.02 ^b^	27.37 ± 0.01 ^d^
PUFA	52.29 ± 0.07 ^d^	52.13 ± 0.01 ^c^	51.48 ± 0.01 ^a^	51.60 ± 0.01 ^b^	52.66 ± 0.02 ^f^	52.54 ± 0.01 ^e^	52.71 ± 0.01 ^f^	52.53 ± 0.02 ^e^	52.64 ± 0.02 ^e,f^
UFA	79.57 ± 0.11 ^c^	79.71 ± 0.01 ^c^	79.15 ± 0.03 ^a^	79.37 ^b^ ± 0.05 ^b^	79.55 ± 0.03 ^c^	80.24 ± 0.03 ^e^	79.90 ± 0.03 ^d^	79.68 ± 0.04 ^c^	80.01 ± 0.04 ^d^
Oil extraction yield (%)	32.27 ± 0.48 ^b^	33.22 ± 0.34 ^a,b^	28.36 ± 0.26 ^b^	30.13 ^a,b^ ± 0.98 ^a,b^	34.24 ± 0.29 ^a,b^	33.87 ± 0.41 ^a,b^	33.48 ± 0.41 ^b^	26.56 ± 0.28 ^b^	26.32 ± 0.33 ^a^

Data are the means of three independent experiments ± standard deviations (n = 3). ^a–h^ Values within the same row with different lowercase letters indicate a significant difference at the 0.05 significance level (Tukey HSD).

**Table 4:** Qualitative parameters of oil extracted from unroasted and roasted pumpkin seeds.

Pumpkin Seeds		Acid Value (AV, mg KOH/g)	Peroxide Value (PV, meq O_2_/kg)	Specific Extinction Coefficients		Antioxidant Activity [μmol TE/g of Oil]
				K_232_	K_270_	
Unroasted		3.62 ± 0.04 ^f^	3.82 ± 0.03 ^a^	1.82 ± 0.03 ^a^	0.73 ± 0.02 ^a^	5.44 ± 0.13 ^c^
Roasted						
110 °C	10 min	3.51 ± 0.03 ^f^	9.17 ± 0.21 ^e,f^	2.13 ± 0.05 ^d,e^	0.87 ± 0.05 ^b,c,d^	4.70 ± 0.20 ^a,b^
	10 min with marjoram	3.60 ± 0.03 ^f^	6.44 ± 0.11 ^b^	1.93 ± 0.04 ^a,b^	0.81 ± 0.03 ^a,b^	5.04 ± 0.39 ^b,c^
	30 min	3.21 ± 0.04 ^d,e^	9.29 ± 0.26 ^e,f^	2.16 ± 0.08 ^e^	0.89 ± 0.03 ^b,c,d^	5.26 ± 0.17 ^b,c^
	30 min with marjoram	3.29 ± 0.03 ^e^	6.70 ± 0.12 ^b,c^	1.96 ± 0.06 ^b,c^	0.83 ± 0.02 ^b,c^	5.38 ± 0.13 ^c^
160 °C	10 min	2.90 ± 0.06 ^b^	9.50 ± 0.19 ^f^	2.21 ± 0.05 ^e,f^	0.91 ± 0.04 ^c,d^	4.40 ± 0.11 ^a^
	10 min with marjoram	3.10 ± 0.05 ^c,d^	7.54 ± 0.11 ^c,d^	2.03 ± 0.03 ^b,c,d^	0.87 ±0.05 ^b,c,d^	4.93 ± 0.27 ^a,b,c^
	30 min	2.59 ± 0.07 ^a^	9.89 ± 0.64 ^f^	2.31 ± 0.07 ^f^	0.95 ± 0.01 ^d^	4.41 ± 0.27 ^a^
	30 min with marjoram	3.02 ± 0.04 ^b,c^	8.23 ± 0.42 ^d,e^	2.10 ± 0.02 ^c,d,e^	0.89 ± 0.03 ^b,c,d^	4.99 ± 0.17 ^a,b,c^

Data are the means of three independent experiments ± standard deviations (n = 3). ^a–f^ Values in the same column with different lowercase letters indicate a significant difference at the 0.05 significance level (Tukey HSD).

**Table 5:** Color profile of oil extracted from unroasted and roasted pumpkin seeds.

Pumpkin Seeds		Total Carotenoid Content (mg/kg)	Total Chlorophyll Content (mg/kg)	L*	a*	b*	∆E
Unroasted		0.76 ± 0.03 ^a^	4.52 ± 0.07 ^f^	42.21 ± 0.90 ^a^	−0.55 ± 0.36 ^d^	22.92 ± 3.12 ^a^	-
Roasted							
110 °C	10 min	3.70 ± 0.04 ^c^	2.41 ± 0.06 ^b^	47.76 ± 1.96 ^a,b,c^	−3.13 ± 0.75 ^b,c^	26.05 ± 0.77 ^a^	7.57 ± 0.66 ^a,b^
	10 min with marjoram	3.38 ± 0.05 ^b^	3.95 ± 0.02 ^e^	44.27 ± 2.24 ^a^	−2.77 ± 1.23 ^c,d^	21.45 ± 1.35 ^a^	4.03 ± 1.67 ^a^
	30 min	4.55 ± 0.07 ^e^	2.53 ± 0.04 ^b^	45.77 ± 2.58 ^a,b^	−2.33 ± 1.18 ^c,d^	26.68 ± 1.91 ^a^	6.23 ± 1.19 ^a,b^
	30 min with marjoram	3.94 ± 0.03 ^d^	3.31 ± 0.05 ^d^	44.79 ± 2.64 ^a,b^	−2.32 ± 1.00 ^c,d^	25.18 ± 1.31 ^a^	5.46 ± 1.30 ^a,b^
160 °C	10 min	3.95 ± 0.07 ^d^	2.15 ± 0.03 ^a^	63.08 ± 1.12 ^d^	−5.97 ± 0.44 ^a^	26.04 ± 0.45 ^a^	21.92 ± 0.77 ^c^
	10 min with marjoram	4.42 ± 0.05 ^e^	3.16 ± 0.04 ^c^	50.84 ± 3.23 ^b,c^	−3.07 ± 0.94 ^b,c^	23.60 ± 1.27 ^a^	9.85 ± 0.44 ^b^
	30 min	3.72 ± 0.06 ^c^	3.96 ± 0.01 ^e^	59.27 ± 1.92 ^d^	−5.41 ± 0.51 ^a,b^	25.67 ± 3.93 ^a^	17.97 ± 1.87 ^c^
	30 min with marjoram	4.02 ± 0.08 ^d^	3.19 ± 0.02 ^c,d^	52.67 ± 2.12 ^c^	−1.90 ± 0.53 ^c,d^	23.05 ± 4.05 ^a^	10.59 ± 0.48 ^b^

Data are the means of three independent experiments ± standard deviations (n = 3). ^a–e^ Values in the same column with different lowercase letters indicate a significant difference at the 0.05 significance level (Tukey HSD).

## Data Availability

The original contributions presented in the study are included in the article, further inquiries can be directed to the corresponding author.
